# Distribution and Mobility of Amines Confined in Porous Silica Supports Assessed via Neutron Scattering, NMR, and MD Simulations: Impacts on CO_2_ Sorption Kinetics and Capacities

**DOI:** 10.1021/acs.accounts.3c00363

**Published:** 2023-09-18

**Authors:** Hyun June Moon, Jan Michael Y. Carrillo, Christopher W. Jones

**Affiliations:** † School of Chemical & Biomolecular Engineering, Georgia Institute of Technology, Atlanta, Georgia 30332, United States; ‡ Center of Nanophase Materials Sciences, Oak Ridge National Laboratory, Oak Ridge, Tennessee 37830, United States

## Abstract

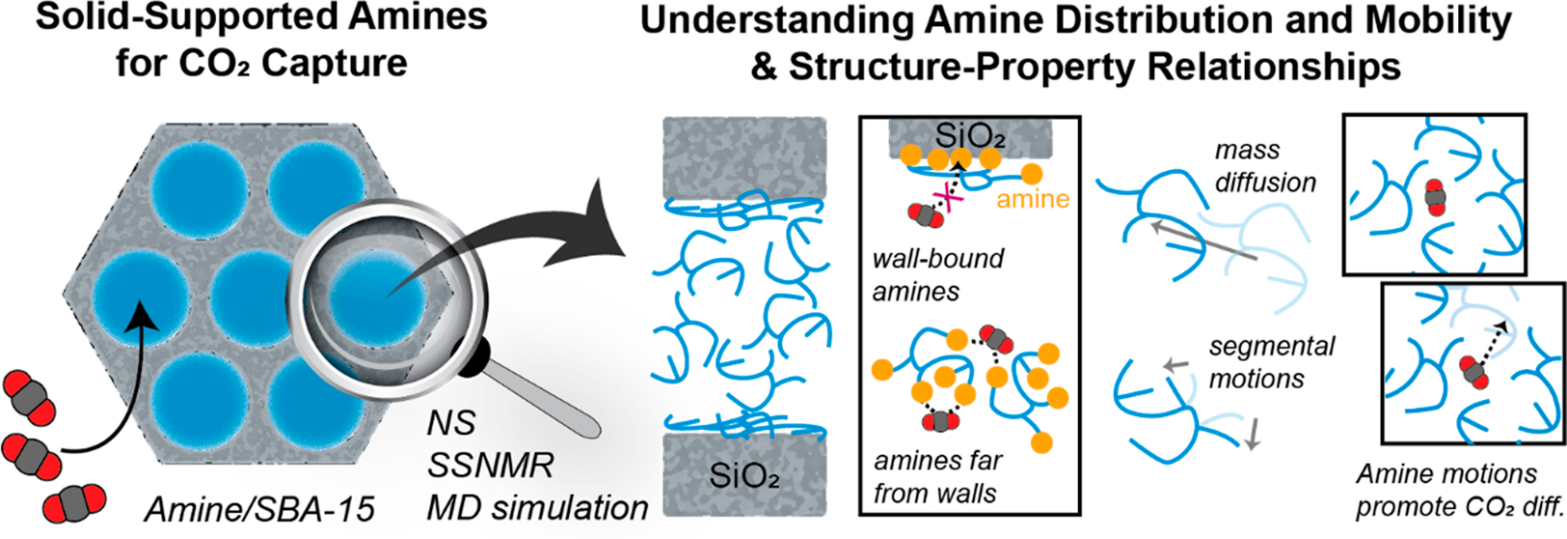

Solid-supported amines are a promising class of CO_2_ sorbents capable of selectively capturing CO_2_ from diverse sources. The chemical interactions between the amine groups and CO_2_ give rise to the formation of strong CO_2_ adducts, such as alkylammonium carbamates, carbamic acids, and bicarbonates, which enable CO_2_ capture even at low driving force, such as with ultradilute CO_2_ streams. Among various solid-supported amine sorbents, oligomeric amines infused into oxide solid supports (noncovalently supported) are widely studied due to their ease of synthesis and low cost. This method allows for the construction of amine-rich sorbents while minimizing problems, such as leaching or evaporation, that occur with supported molecular amines.

Researchers have pursued improved sorbents by tuning the physical and chemical properties of solid supports and amine phases. In terms of CO_2_ uptake, the amine efficiency, or the moles of sorbed CO_2_ per mole of amine sites, and uptake rate (CO_2_ capture per unit time) are the most critical factors determining the effectiveness of the material. While structure–property relationships have been developed for different porous oxide supports, the interaction(s) of the amine phase with the solid support, the structure and distribution of the organic phase within the pores, and the mobility of the amine phase within the pores are not well understood. These factors are important, because the kinetics of CO_2_ sorption, particularly when using the prototypical amine oligomer branched poly(ethylenimine) (PEI), follow an unconventional trend, with rapid initial uptake followed by a very slow, asymptotic approach to equilibrium. This suggests that the uptake of CO_2_ within such solid-supported amines is mass transfer-limited. Therefore, improving sorption performance can be facilitated by better understanding the amine structure and distribution within the pores.

In this context, model solid-supported amine sorbents were constructed from a highly ordered, mesoporous silica SBA-15 support, and an array of techniques was used to probe the soft matter domains within these hybrid materials. The choice of SBA-15 as the model support was based on its ordered arrangement of mesopores with tunable physical and chemical properties, including pore size, particle lengths, and surface chemistries. Branched PEI—the most common amine phase used in solid CO_2_ sorbents—and its linear, low molecular weight analogue, tetraethylenepentamine (TEPA), were deployed as the amine phases. Neutron scattering (NS), including small angle neutron scattering (SANS) and quasielastic neutron scattering (QENS), alongside solid-state NMR (ssNMR) and molecular dynamics (MD) simulations, was used to elucidate the structure and mobility of the amine phases within the pores of the support. Together, these tools, which have previously not been applied to such materials, provided new information regarding how the amine phases filled the support pores as the loading increased and the mobility of those amine phases. Varying pore surface-amine interactions led to unique trends for amine distributions and mobility; for instance, hydrophilic walls (i.e., attractive to amines) resulted in hampered motions with more intimate coordination to the walls, while amines around hydrophobic walls or walls with grafted chains that interrupt amine-wall coordination showed recovered mobility, with amines being more liberated from the walls. By correlating the structural and dynamic properties with CO_2_ sorption properties, novel relationships were identified, shedding light on the performance of the amine sorbents, and providing valuable guidance for the design of more effective supported amine sorbents.

## Key References

- HolewinskiA.; Sakwa-NovakM. A.; JonesC. W.Linking CO_2_ sorption performance to polymer morphology in aminopolymer/silica composites through neutron scattering. J. Am. Chem. Soc.2015, 137, 11749–117592630818310.1021/jacs.5b06823.^1^*This article illustrates how small-angle neutron scattering (SANS) was used as an effective tool for characterizing PEI morphologies in SBA-15 and the impacts of the morphologies on CO_2_ uptake performance.*
- HolewinskiA.; Sakwa-NovakM. A.; CarrilloJ.-M. Y.; PotterM. E.; EllebrachtN.; RotherG.; SumpterB. G.; JonesC. W.Aminopolymer mobility and support interactions in silica–PEI composites for CO_2_ capture applications: A quasielastic neutron scattering study. J. Phys. Chem. B2017, 121, 6721–67312855820910.1021/acs.jpcb.7b04106.^2^*This article demonstrates that QENS is a robust method for quantitatively analyzing diffusive dynamic properties of mesopore confined PEI and the effect of the attraction of the PEI toward the pore walls*.
- MoonH. J.; CarrilloJ.-M. Y.; LeisenJ.; SumpterB. G.; OstiN. C.; TyagiM.; JonesC. W.Understanding the impacts of support-polymer interactions on the dynamics of poly(ethylenimine) confined in mesoporous SBA-15. J. Am. Chem. Soc.2022, 144, 11664–116753572977110.1021/jacs.2c03028.^3^*This article presents combining neutron scattering and solid-state NMR studies to yield comprehensive understanding of polymer mobility in SBA-15 under varied wall–polymer interaction conditions*.
- MoonH. J.; SekiyaR.; JonesC. W.Probing the morphology and mobility of amines in porous silica CO_2_ sorbents by ^1^H T_1_-T_2_ relaxation correlation NMR. J. Phys. Chem. C2023, 127, 11652–11665.^4^*This article describes that ^1^H relaxation time measurements can capture morphology and mobility of amines with different chain topologies as well as varied pore fill fraction.*

## 1 Introduction

Solid-supported amines are among the most widely studied CO_2_ adsorbents and are the backbones of many emerging CO_2_ separation applications. Many types of solid supported amines exist—MOFs with pendant amines tethered to organic ligands or metal centers,^5−9^ porous polymers or COFs containing backbone or pendant amines,^10−13^ as well as porous solid supports on which (or in which) amines are incorporated.^14−17^ One widely studied class of supported amine materials is mesoporous oxide supports (i.e., silica, alumina, mixed oxides, etc.) functionalized with organic moieties containing alkyl amine groups.^15,17−22^ With these supports, amines can be either covalently bound to the pore surfaces^15,18−22^ or physically contained in the pores.^17^ While covalent bonding yields excellent stability to moderate temperature swings and exposure to humidity, physical confinement of amines enables simpler syntheses and often, higher CO_2_ uptakes due to higher amine loadings compared with sorbents containing only chemically grafted amines (for grafting, amine loading scales with surface area, whereas for amine impregnation, amine loading scales with mesopore volume),^23^ with potential limitations around leaching or evaporation of amines under suboptimal conditions. These sorbents are often used in temperature swing adsorption (TSA) cycles or temperature vacuum swing adsorption (TVSA) cycles, making the volatility of the amine component an important parameter for sorbents based on noncovalent bonding of amines to supports. Indeed, many classical amine solvents (e.g., monoethanolamine, diethanolamine, etc.) and low molecular weight amine oligomers (e.g., triethylenetetramine (TETA), tetraethylenepentamine (TEPA), etc.) are likely too volatile to be effectively deployed in solid sorbents at ambient and higher temperatures, despite numerous academic studies exploring such materials.

**Figure 1:**
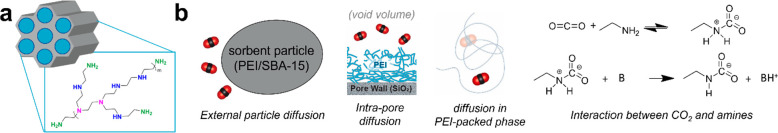
Schematic structure and the CO_2_ capture mechanism in PEI/SBA-15. (a) Schematic showing PEI/SBA-15. (b) CO_2_ diffusion pathways reaching amine groups and interaction with amines resulting in CO_2_ sorption.^16,42,43^ CO_2_–amine interaction reprinted with permission from ref (16). Copyright 2011 Royal Society of Chemistry.

A representative example of this type of supported amine sorbent is branched poly(ethylenimine) (PEI) (MW ∼ 800 g/mol) physically impregnated into the pores of ordered mesoporous SBA-15 silica (PEI/SBA-15) (Figure 1a). While more scalable, practical sorbents use disordered mesoporous supports, ordered supports like SBA-15 and MCM-41 facilitate fundamental scientific investigations, as described in this article. Song and co-workers^17^ discovered that inclusion of aminopolymers in porous silica supports exhibited promising CO_2_ uptake performance. However, they observed an unusual, antithermodynamic behavior of the materials, where CO_2_ uptake initially increased with temperature before reducing upon further heating. The authors attributed this to diffusion limitations imposed by the polymer at low temperatures that were alleviated by adding heat to the system. When polymer chains were given sufficient mobility to allow for suitable CO_2_ mass transfer, a further temperature increase resulted in the expected thermodynamic behavior. Thus, from the earliest studies on these materials, the mobility of the supported amines within the mesopores of the support was recognized as an important factor. Further research on those materials aimed to improve their CO_2_ uptake properties by varying pore sizes,^24−26^ leaving behind surfactants (e.g., CTAB)^24−26^ around pore walls, using additives (e.g., PEG)^27−29^ or smaller, molecular amines (e.g., diethanolamine),^30,31^ and using combinations of grafted amines together with physically impregnated amines.^32,33^ Researchers observed varied gas capture performance under these different circumstances. In many cases, the authors suggested that a more favorable distribution and motions of amines were the main driving forces behind the improved performance.

During CO_2_ capture on such supported amines, CO_2_ molecules initially enter the pore openings of the solid materials, diffuse through the available free volume, reach amine sites, and then interact with the amines through adsorption or chemical reaction (Figure 1b).^34^ As CO_2_ adsorption consumes amine sites, CO_2_ must diffuse through the amine compounds within the pores to access additional free amine sites. However, this diffusion process is often slower due to mass transfer barriers imposed by the amine polymer. Studies of the dynamics of CO_2_ adsorption on these materials show two distinct sorption regimes; an initial rapid phase followed by a slower approach to pseudoequilibrium.^35^ When CO_2_ reacts with amine groups under dilute (≤10% CO_2_) or ultradilute conditions (≤1% CO_2_), it typically reacts to form carbamic acid species (secondarily) and alkyl ammonium carbamates (primarily).^17,18^ The formation of carbamates involve the deprotonation of a carbamic acid species by an adjacent amine.^36,37^ Carbamates are thermodynamically favored, and most amine sorbents that display high CO_2_ uptake capacities adsorb CO_2_ primarily as carbamates. Carbamate formation has the potential to cross-link amine chains due to the amine:CO_2_ stoichiometry (2:1), which can rigidify the organic phase. This phenomenon has been hypothesized to be a key driver of the slow approach to equilibrium observed in experimental studies.^38−40^ Under humid conditions, CO_2_ uptakes often improve, and this has been associated with an additional CO_2_ adsorption manifold, bicarbonate formation,^41^ which has 1:1 amine:CO_2_ stoichiometry, and also to enhanced polyamine mobility due to water disrupting hydrogen bonding networks. One gauge of the efficiency of these materials is amine efficiency (AE), defined as the amount of CO_2_ captured per total amine loading, typically represented by mmol of CO_2_/ mmol of N. The interactions between CO_2_ and amines mentioned above lead to the theoretical AE maxima ranging from 0.5 (when forming ammonium carbamate) to 1.0 (bicarbonate or carbamic acid).

Supported amines typically exhibit limited AE, reaching up to ∼0.2 under dry 400 ppm of CO_2_, simulating DAC.^23,44^ This value is far from the theoretical maxima (0.5). Rationalizing these behaviors requires knowledge of the local structure of the polyamines within the support pores as well as insight into amine mobility, including center of mass diffusion, local chain mobility, and bond dynamics. Unfortunately, conventional laboratory characterization techniques for these hard/soft composite materials predominantly provide information about the hard oxide domains. The PEI used in these materials is low molecular weight and exhibits a honeylike consistency under ambient conditions. While researchers have systematically altered key structural aspects of the support such as average support pore diameter, support pore volume and support channel length and particle size,^45−48^ limited insight is available regarding the structure and dynamics of the supported, low MW branched PEI phase. Some empirical knowledge has been gathered correlating CO_2_ uptake behavior to the structure of aminopolymers with varied structures (e.g., branched vs linear, MW, grafted PEI),^49−52^ but little is known about how these polymers fill the pores or move within the pores. To address this gap, we employed techniques from the soft matter community to investigate supported amine adsorbents with the goal of directly probing the polymer domains in these composite materials.

## 2 Model Supported Amine CO_2_ Sorbents and Techniques to Understand Physical Properties of Sorbent Materials

There are numerous practical and scalable supported amine CO_2_ sorbents that utilize mesoporous oxide supports with a limited structural order. However, to understand the structural and dynamic properties of supported amines, well-defined support materials are necessary. SBA-15 silica consists of regular arrays of mesopores, and literature synthesis protocols were used to ensure well-controlled mesopore sizes and particle morphologies.

Direct characterization of solid-supported PEI can be effectively achieved using experimental tools, such as neutron scattering (NS) and solid-state NMR (ssNMR). To aid in the interpretation of the experimental findings, we utilized coarse-grained molecular dynamics (MD) simulations. These approaches enable characterization of various important PEI properties, as listed in Table 1. Detailed discussions about the techniques and methods can be found in the following examples.

**Table 1:** Approaches Used for Understanding PEI Properties within Mesoporous Silica

Approach	Characteristics	Accessible PEI properties	Scales (length, time)
Small-angle neutron scattering	Penetrable, contrast (PEI vs silica)	Morphology	0.1–1000 nm^53,54^
Quasielastic neutron scattering	Penetrable, selective (PEI mainly)	Mobility (center-of-mass or segmental diffusion)	1 ps–1 ns^55,56^
Solid-state NMR (^1^H relaxation)	Penetrable, selective (isotopes, spins)	Morphology^a^ and mobility^a^	0.1–100 nm^57^
			ns–ms^42^
MD simulation	Predict PEI behavior, crosscheck NS and ssNMR results	Morphology and mobility	0.1–100 nm
			1 ps–1000 ns^58^

a ssNMR yields qualitative information and trends, whereas NS and MD simulation give quantitative results.

## 3 Distribution and Mobility of Amines Supported in SBA-15 Explored via NS, ssNMR, and MD Simulation

### 3.1 Structure and Distribution of PEI in Mesoporous SBA-15 Silica

The limited AE of PEI/SBA-15 systems may originate from the physical properties of confined amines, and one contributing factor may be the unfavorable distribution of amines within the mesopores. Branched PEI contains different types of amine groups, with primary amines dispersed in the peripheral region of PEI domains and tertiary amines hidden in the core. These domains, containing hydrophilic and basic amine groups, may strongly coordinate with the hydrophilic parts of the pore walls of the solid support. SBA-15 surfaces contain slightly acidic silanol groups, suggesting possible amine-silanol interactions that attract PEI toward the pore walls. This attraction may result in loss of the ability of wall-coordinating amine groups to capture CO_2_. Our observations support this interpretation, as PEI/SBA-15 with low amine loadings showed limited AE (∼0.05 mol CO_2_/mol N), whereas sorbents with intermediate or high amine loadings yielded much higher AE (0.1–0.15), with a smaller fraction of total PEI anchored to the pore walls.^1,2,4^ Therefore, we assert that amines closely located to the support surface are not effective sorption sites for CO_2_ capture, and the amount of available active amines is governed by the morphology of PEI within the support. Hence, understanding the amine structure and morphology at different amine loadings is crucial.

Small-angle neutron scattering (SANS) is a technique well-suited for probing the structural properties of PEI/SBA-15 systems for two key reasons. First, neutrons can effectively pass-through condensed silica matter and reach the soft PEI.^53^ Second, we can leverage differences in the effectiveness of interactions with incident neutrons, known as “contrast,” to enhance scattering from specific domains. By using deuterated PEI (dPEI), a significant difference in neutron scattering length density (SLD) can be achieved (dPEI ∼ 8.2, SiO_2_ ∼ 3.5, units: 10^–6^ Å^–2^).^59^ Maximizing coherent neutron scattering^53,60,61^ through deuteration also helps acquire SANS spectra with suitable signal-to-noise ratio.

**Figure 2:**
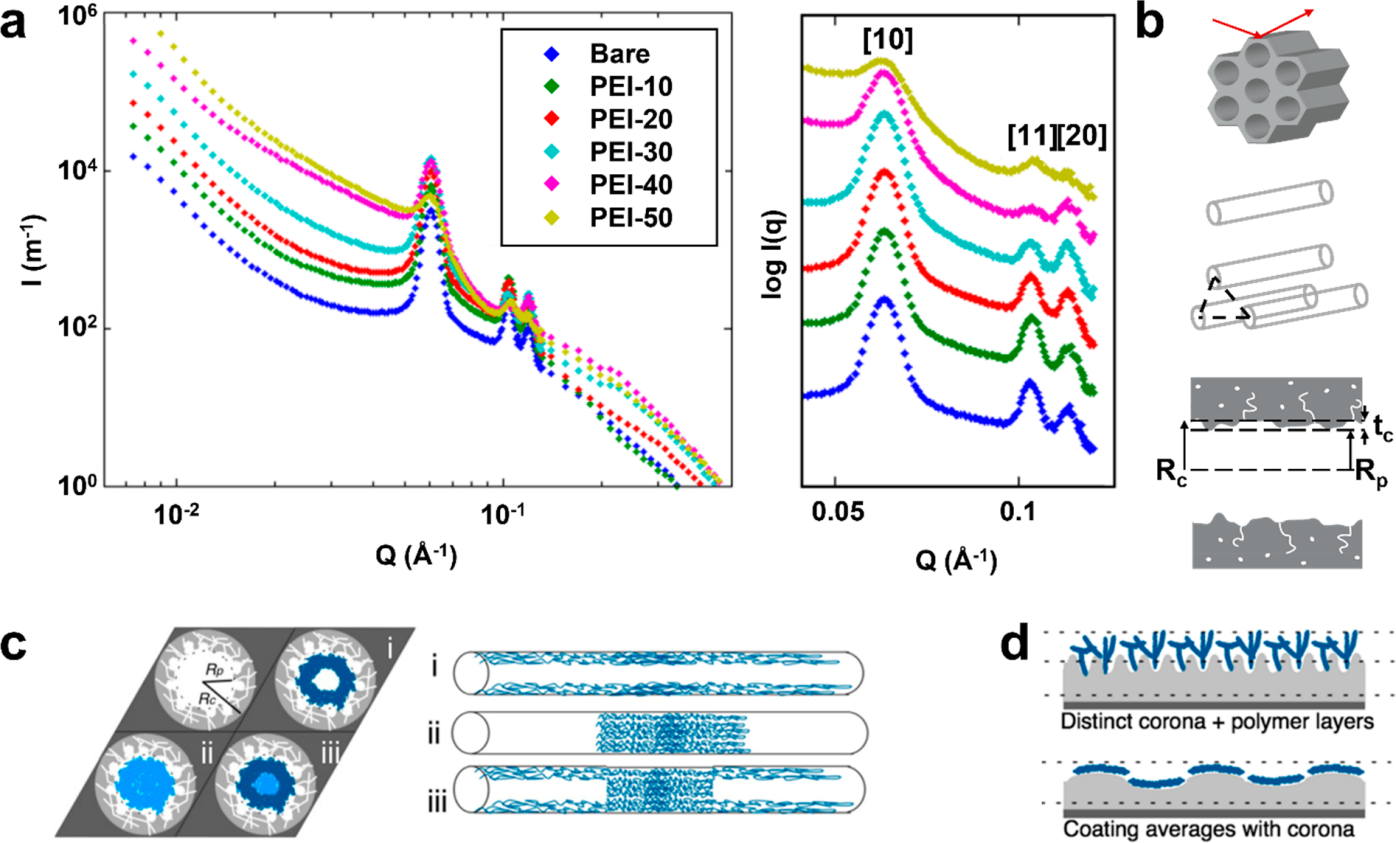
Structural characteristics of PEI/SBA-15 captured via SANS. (a) SANS spectra (intensity vs scattering vector Q) of bare SBA-15 support and PEI/SBA-15 composites with varied PEI loadings, with diffraction peaks highlighted. (b) Key structural features captured via SANS, such as particle surface scatter, form factor of a mesopore, structure factor showing arrangement of mesopores, and structural inhomogeneities.^53,62^ (c) PEI morphologies considered, where i represents consistent PEI deposition on the pore walls, ii represents formation of PEI plugs in the pores, and iii indicates mixed cases of the formers. (d) Possible PEI morphologies around the wall–PEI interfaces with different extents of surface roughness. Particle length of SBA-15 is approximately 1 μm.^1^ Reproduced with permission from ref (1). Copyright 2015 American Chemical Society.

We sought to understand how PEI fills the mesopores of SBA-15 and used neutron scattering to generate this understanding. SANS spectra of dPEI/SBA-15 with varying amounts of dPEI were obtained. Upon introduction of dPEI to the silica mesopores, noticeable changes were observed in the ratios of the silica diffraction peaks [10], [11], and [20]. These changes were attributed to changes of the SLD distribution around the wall-polymer interface and within the previously evacuated pore spaces (Figure 2a). The spectra were subsequently compared to theoretical models, considering various contributions, such as particle surface scatter (Porod’s law), the form factor of a single mesopore, a structure factor that is related to the arrangement of repeated structures of the mesopores, and diffuse scattering from structural inhomogeneities (Figure 2b). This analysis allowed the estimation of key structural aspects such as (i) morphology of PEI within mesopores of SBA-15, (ii) the characteristic lengths of different domains, and (iii) the probable structures of PEI around the wall-polymer interface.

We first focused on understanding how PEI fills the mesopore space. Three different types of models were considered (Figure 2c)—wall coating dominant phases, plug dominant phases, and mixed cases. Among these morphologies, the best fits were associated with PEI initially forming conformal coating layers (referred to as pore-coating PEI) around the pore walls, occupying up to ∼20 vol % of the pore. As more PEI was added, it subsequently formed aggregates within the pores. By analysis of the characteristic lengths of the pore-coating and aggregate PEI domains, SANS curve fitting yielded plausible thicknesses of these domains, with the pore-coating thickness roughly 10–15 Å. Furthermore, by calculating the SLD values of the wall–PEI interfaces (i.e., called the “corona,” associated with silica surface roughness, Figure 2d), it was possible to estimate the likely structure of PEI around the wall–PEI interfaces. In supports with relatively rough surfaces, a clear increase in the SLD was observed due to the formation of a PEI-rich layer (Figure 2d, upper case). On the other hand, SBA-15 with relatively smooth pore surfaces (Figure 2d, lower case) had relatively lower SLD values, indicating a lower volume fraction of PEI at the interfaces compared to rough surfaces.^38^

### 3.2 Mobility of Confined PEI in SBA-15 and Effects of Wall–PEI Interactions^2,3^

Attractive interactions between the pore wall and polymer may also affect amine mobility, which can further affect the CO_2_ diffusivity. To probe PEI mobility, we conducted quasielastic neutron scattering (QENS) experiments on PEI/SBA-15 systems with two distinctly different types of PEI–wall interactions. In one system, the pore walls possessed native silanol groups, which are slightly acidic and strongly interact with the basic amine groups of PEI. In the second system, the pore surfaces were capped with hexamethyldisilazane (HMDS), resulting in the presence of trimethylsilyl groups that repel the hydrophilic PEI end groups. QENS enabled us to experimentally explore the dynamics of PEI, with incoherent neutron scattering providing data that could be analyzed to determine self-correlation functions related to the dynamics of the PEI. Hydrogen has a significantly larger extent of incoherent scattering compared to other nuclei present in PEI/SBA-15 systems (i.e., C, N, Si, and O), and therefore, using PEI with the usual natural abundance of ^1^H (∼99.99%) was beneficial to the study of PEI dynamics.

**Figure 3:**
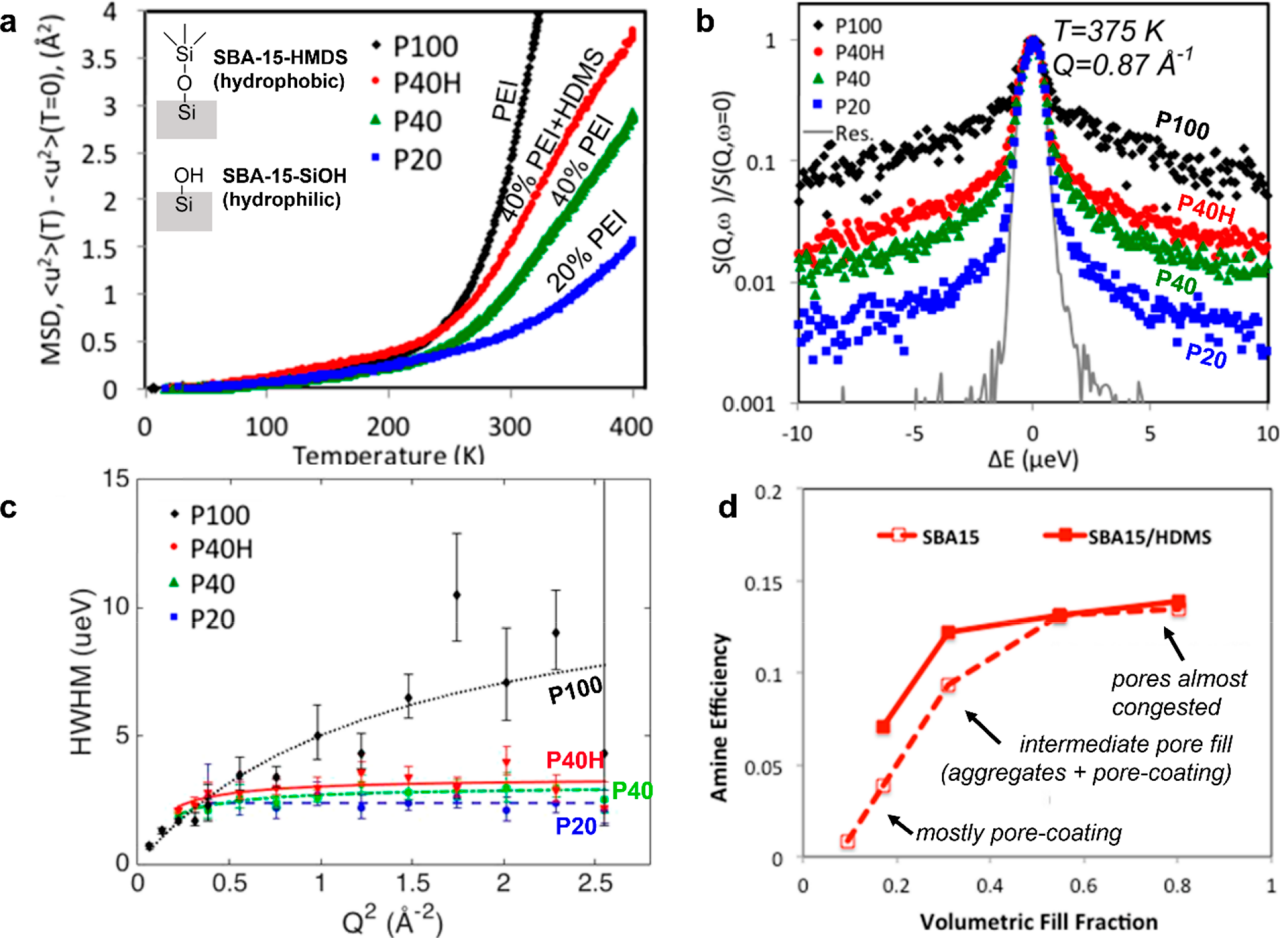
Characterization of PEI mobility using QENS and impacts on CO_2_ uptake. (a) Mean-square displacement as a function of temperature taken by QENS, showing trend of PEI mobility. (b) QENS spectra taken at 375 K with the momentum transfer (Q) of 0.87 Å^–1^ (normalized intensities vs energy transfer). (c) QENS spectral widths (half-width at half-maximum; HWHM) fitted against jump-mediated diffusion model. (d) Amine efficiencies as a function of volumetric fill fraction of PEI/SBA-15, showing relationship between PEI mobility and CO_2_ uptake.^2^ Reproduced with permission from ref (2). Copyright 2017 American Chemical Society.

Different amounts of PEI were loaded in SBA-15 targeting sorbent materials with different fractions of pore-coating and aggregate PEI. Both native silanol-containing walls and trimethylsilyl-capped supports were used, producing different wall–PEI interactions. QENS experiments were conducted in two modes: first, yielding the mean-square displacement (MSD) as a function of temperature (Figure 3a) and then producing QENS spectra (Figure 3b). The notation used here includes P100 for nonconfined free PEI, P20 representing a composite with 20 wt % PEI mostly representing wall-coating PEI, P40 denoting a composite with 40 wt % PEI consisting of both aggregates and wall-coating PEI, and P40H indicating a composite with 40 wt % PEI and hydrophobized pore walls.

The MSD as a function of temperature shown in Figure 3a shows a greater extent of diffusive motions (i.e., larger MSD at given temperatures) for PEI as the sorbents are loaded with larger amounts of PEI aggregates (P20 vs P40) and under conditions with reduced attraction to the pore walls (P40 vs P40H). Consistent with the MSD versus temperature plot, the QENS spectra (Figure 3b) showed varying degrees of broadening for different materials (where broader spectra indicate faster motions), which are related to the different diffusive mobilities of PEI in each case. Figure 3c demonstrates extracted spectral widths that were fitted to a jump-mediated diffusion model, eventually yielding dynamic parameters, such as the time scale, jump lengths, and diffusivity (Table 2). PEI mobility was shown to correlate with the effectiveness of the CO_2_ sorbents. It was observed that improved PEI mobility yielded a better AE (Figure 3d). Once the pore fill fraction reached a certain extent, further improved PEI mobility did not yield better CO_2_ sorption performance, probably due to limited CO_2_ diffusivity through PEI aggregates located far from the pore walls.

**Table 2:** Characteristics of Center-of-Mass Diffusive Motions of PEI^2^

	⟨*L*^2^⟩^1/2^ (Å)			τ (ns)			*D* (×10^–11^ m^2^/s)		
*T* (K)	325	350	375	325	350	375	325	350	375
P100 (bulk PEI)	3.7	2.0	2.1	0.29	0.09	0.06	7.0	7.0	7.0
P20 (20 wt % PEI)			3.2			0.22			9.4
P40 (40 wt % PEI)	6.9	6.6	7.9	0.26	0.23	0.22	32	32	32
P40H (40 wt % PEI)	9.2	4.3	6.5	0.27	0.17	0.19	3.8	0.53	1.3

Reproduced with permission from ref (2). Copyright 2017 American Chemical Society.

**Figure 4:**
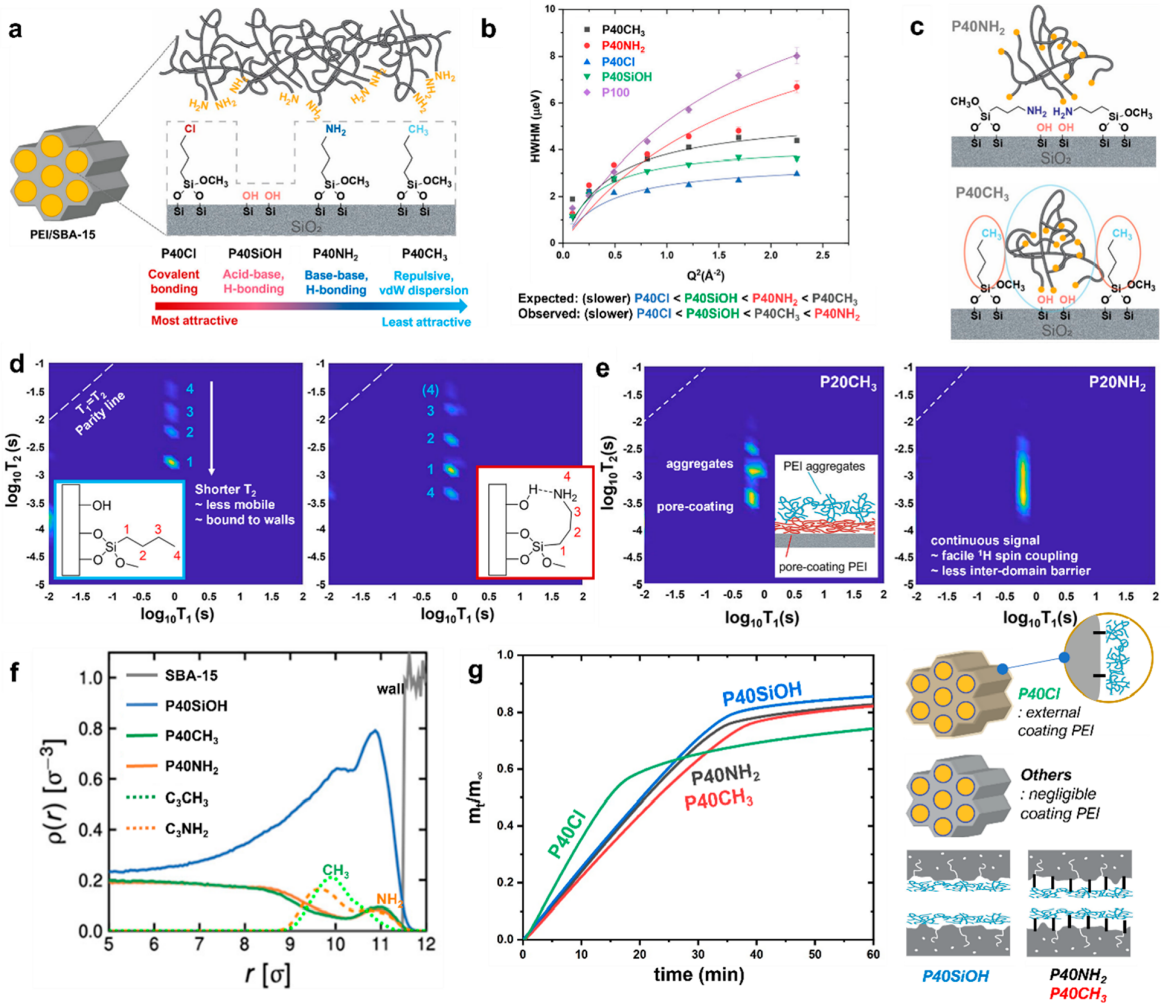
Impacts of wall–PEI interactions in PEI mobility and distribution. (a) Different types of wall–PEI interactions. (b) Trend of PEI mobility understood by analyzing the spectral widths of QENS spectra (curve fitting done by jump-mediated diffusion). (c) Hypothesized configurations of wall-grafted alkylamine chains and alkyl chains and PEI distribution around pore walls. (f) ^1^H T_1_–T_2_ correlation plots for wall-grafted chains. (e) ^1^H T_1_–T_2_ plots for PEI around pore walls. (f) Density distribution function calculated by MD simulation (probability vs distance, where *r* ∼ 11 refers to the walls), where dotted lines represent wall-grafted chains and solid lines represent primary amine groups of PEI. (g) Comparison of CO_2_ uptake rates based on fractional uptake versus time and underlying causes. Reproduced with permission from ref (3). Copyright 2022 American Chemical Society.

We further diversified the range of PEI–wall interactions and investigated the impact of different silica surface treatments on the PEI mobility. Silanes with varying end groups were grafted on the pore walls to modulate the PEI–wall interactions (Figure 4a). The mobility of PEI in 40 wt % PEI loaded composites (P40Cl, P40SiOH, P40NH_2_, P40CH_3_) was probed by QENS, where we hypothesized that less attractive PEI–wall interactions would give rise to faster PEI motions (i.e., P40Cl showing the slowest motions, as this functional group can form covalent bonds with the PEI, followed by P40SiOH, P40NH_2_, with P40CH_3_ giving the fastest motions). Upon analysis of QENS spectra, this hypothesis was refuted (Figure 4b). P40NH_2_ displayed faster motions than P40CH_3_, for example. The data led to a new hypothesis that the conformation of the wall-grafted chains may differ depending on the end groups (e.g., hydrophilic NH_2_ vs hydrophobic CH_3_) (Figure 4c). This hypothesis was tested by ssNMR ^1^H T_1_–T_2_ relaxation correlation measurements from which we determined plausible chain configurations for the wall-grafted chains (Figure 4d). The proton relaxation correlation for the PEI domains close to the silica support walls also supported the hypothesized structures. T_1_–T_2_ plots for 20 wt % PEI, which represent PEI around the pore walls, suggested that alkylamine groups on the walls effectively cap hydrophilic area of the walls, blocking PEI–wall interactions and creating a continuous distribution of T_2_ (i.e., effective ^1^H spins’ dipolar coupling) (Figure 4e). On the other hand, hydrophobic chains grafted on the walls did not coordinate closely to hydrophilic residues of the walls, and PEI–wall interactions were less affected compared to P20NH_2_. That in turn created an energy penalty when PEI located close to walls (and surrounded by hydrophobic alkyl chains) moved diffusively. The discontinuous T_2_ signal contributions suggested the existence of diffusion barriers in the PEI domains adjacent to the silica walls (Figure 4e). Additionally, the density distribution calculated by MD simulations reflecting the interplay between wall-grafted chains and the silica surface (including hydrophilic portions such as silanols) captured a similar trend, as shown in the T_1_–T_2_ NMR (Figure 4f, *dotted lines*). The solid lines representing the population of periphery primary amines of branched PEI showed subtle differences in the P40CH_3_ and P40NH_2_ materials at *r* ∼ 11σ, suggesting that the PEI distribution was also affected (Figure 4f, *solid lines*).

The effects of PEI mobility were then interpreted in terms of the CO_2_ uptake rates (Figure 4g). The data show that the macroscopic structures described above affected the initial uptake rates. The presence of PEI coating the external surface of the SBA-15 particles rapidly captured CO_2_ (Figure 4g, *P40Cl*), and faster uptake rates were observed for the sorbents that had more free volume within the mesopores (Figure 4g, *P40SiOH* vs *P40CH*_*3*_ and *P40NH*_*2*_). For materials with similar macroscopic structures and extents of free volume in the mesopores, the CO_2_ uptake was faster in the sorbents having faster PEI mobilities (Figure 4g, *P40CH*_*3*_ vs *P40NH*_*2*_).

### 3.3 Behavior of Supported Amines as a Function of Chain Topologies^4^

The literature shows that oligomeric or polymeric amines with different chain topologies show distinct CO_2_ capture behavior. Among widely used amines, TEPA and PEI share similar chemical identities (i.e., amine groups separated by ethylene spacers) but exhibit discernible behavior in CO_2_ sorption when supported in SBA-15. TEPA generally shows faster uptake but less stability of repeated cycling. These differences are ascribed to their unique chain topologies. TEPA has short (MW ∼ 200 g/mol) and relatively linear structures,^63,64^ whereas PEI has larger MW (∼800 g/mol) with highly branched structures.

**Figure 5:**
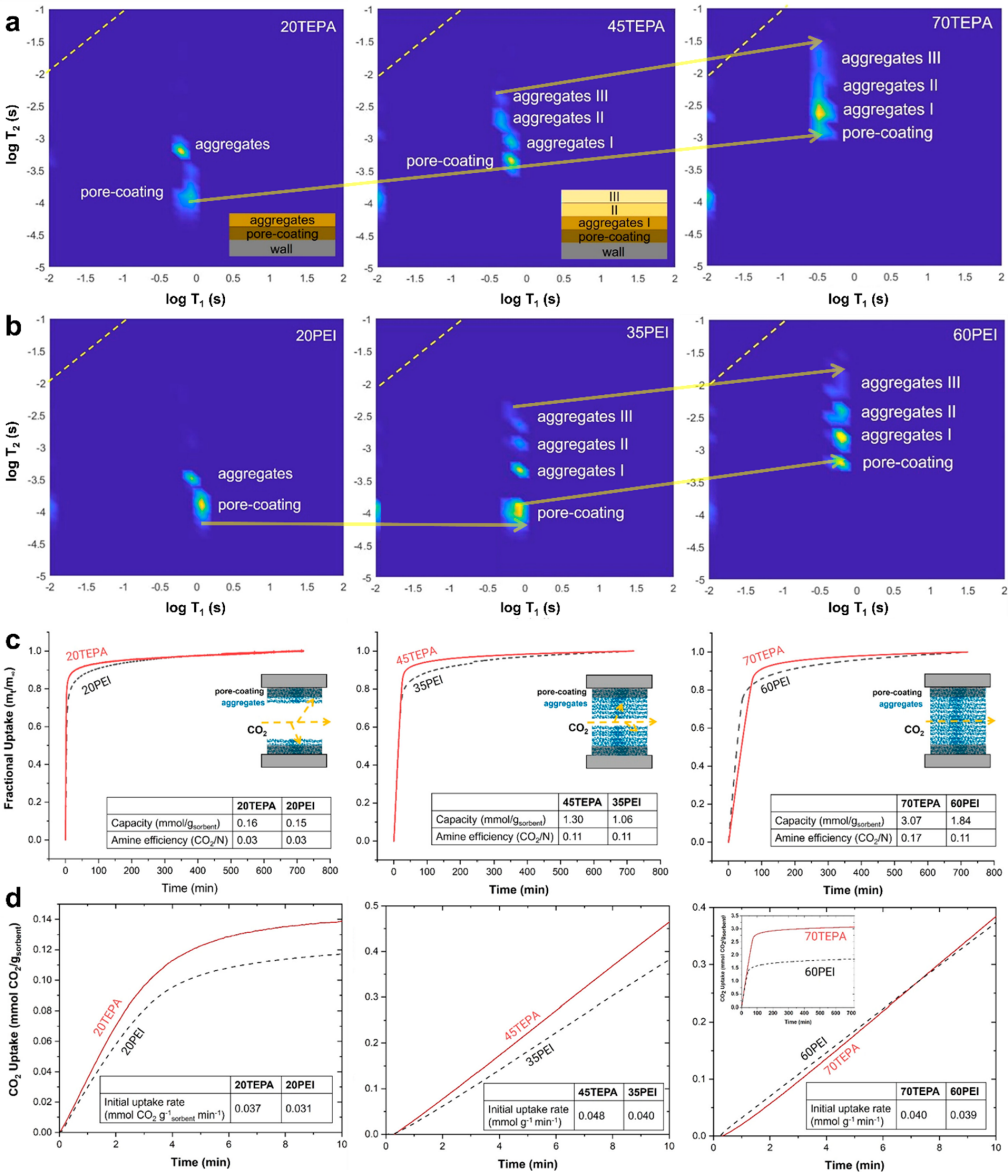
Morphology and mobility of amines with different chain topologies probed by ^1^H T_1_–T_2_ NMR and relationship to CO_2_ sorption behavior. (a) ^1^H T_1_–T_2_ plots for TEPA/SBA-15 with varied loadings. (b) T_1_–T_2_ plots for PEI/SBA-15 with matching pore fill fraction to TEPA cases. (c) CO_2_ sorption results shown by fractional uptake, pseudoequilibrium capacities, and amine efficiencies. (d) CO_2_ uptake curves at early stages and the initial uptake rates calculated in the semilinear region approximately 0–10 min.^4^ Reproduced with permission from ref (4). Copyright 2023 American Chemical Society.

To probe the structure and mobility of TEPA and PEI supported in SBA-15 and link their physical properties to their gas sorption behavior, we conducted ^1^H T_1_–T_2_ correlation NMR studies on TEPA/SBA-15 and PEI/SBA-15 with varied weight loadings of amine. Figure 5a and 5b shows the T_1_–T_2_ correlation plots along with expected amine distributions and amine mobility trends. Considering the 20TEPA and 20PEI materials (both ∼20 wt % amines and ∼40% pore filling), TEPA showed a more diffuse signal for the pore-coating domain compared to PEI. These differences can be related to their unique chain topologies. Branched PEI, with multiple flexible arms, sticks to the silica surfaces via multidentate interactions, while the short and linear TEPA likely adopts only monodentate or bidentate binding. When comparing the cases with intermediate pore fill fractions (∼60%; 45TEPA, 35PEI), the signal linked to the pore-coating TEPA showed notably increased T_2_ values with decreased T_1_, suggesting faster mobility. On the other hand, the pore-coating signal appearing in 35PEI did not show significant T_1_ and T_2_ shifts from the lower amine loading case. We suggest that a significant jump in wall-bound TEPA mobility may arise from the interactions between mobile TEPA molecules that are less engaged in wall–TEPA interactions (TEPA aggregates) and TEPA on the walls. The data suggest that mobile TEPA can at least partly detach wall-bound TEPA chains and increase the mobility of the wall domains. However, PEI molecules having multiple arms bound to the walls may have a larger barrier to such enhanced mobility. Lastly, when considering the composites almost completely loaded with amines (70TEPA and 60PEI, pore fill fraction ∼100%), a significant jump in molecular mobility in both the TEPA and PEI was observed. This unexpected finding suggests that pores that are nearly filled with amines give enhanced spin coupling and spin diffusion, indicative of enhanced molecular mobility.

The qualitative information about amine distribution and dynamics taken from NMR was then correlated to the CO_2_ uptake of the sorbents. Figure 5c shows the fractional CO_2_ uptake curves and AEs. The cases with lower amine content (20TEPA and 20PEI) showed considerably lower AE compared to other cases. This can be explained as noted above, with pore-coating layers resulting in buried amines toward the pore walls.^1^ Compared with low amine loadings, materials with intermediate and high amine loadings (45–70TEPA and 35–60PEI) showed much higher AE, presumably caused by more PEI in aggregates. In the case of PEI, further increases in the aggregate content (60PEI, pore-occluded case) did not return better AE. In this case, CO_2_ mainly diffuses through fully amine-packed pores, where the formation of ammonium carbamate further reduced the molecular mobility of PEI by cross-linking amine groups. In contrast, 70TEPA showed a remarkably larger AE, likely due to the greater extent of molecular mobility and less pronounced effect of CO_2_-induced cross-linking. Lastly, Figure 5d shows CO_2_ uptake curves over the early sorption period and initial uptake rates. Comparing lower and intermediate loadings, we found that faster amine mobility correlates to faster CO_2_ uptake. However, sorbents fully loaded with amines showed slower uptake due to the congestion of the pores.

### 3.4 Prediction of Molecular Behavior of Amines via MD Simulation^65^

To aid in the interpretation of the NS and ssNMR results mentioned earlier, we used coarse-grained molecular dynamics simulations at the bead–spring level. In this approach, a single Lennard-Jones bead represents a PEI monomer, and the interactions among beads are chosen to mimic hydrophobic and hydrophilic interactions by adjusting the cutoff and the energy well-depth, ε, of the shifted and truncated Lennard-Jones potential, which describes the interaction between a pair of beads. A larger value of ε represents a stronger attraction, while a cutoff at the minimum of the potential represents purely repulsive interactions. The probabilities of selective adsorption of beads toward a bead belonging to the mesoporous support can also be estimated by comparing ε using the Boltzmann factor. The simulation was designed to incorporate a strong attraction between silanols and the primary amines, while the model retains the branched architecture of the PEI.

Despite the primitive nature of the model and the lack of specific chemical details, the simulation approach could describe the structure and dynamics of the adsorbed chain by calculating the density distributions and dynamic structure factors at different length and time scales. This description shows reasonable agreement with the results of SANS and QENS experiments. In particular, the simulations observe the conformal coating and aggregation of the polymers at mesopores, which can be directly linked to the fast and slow segmental dynamics of the chain.^66^ This approach allows for the investigation of the morphology of the support, ranging from cylinders to double gyroids.^65^ By analysis of the dynamics of the chains in the simulation, a correlation between the dynamics of the chain and the mesopore morphology was elucidated.

## 4 Conclusions and Outlook

Solid-supported amines are a promising platform for CO_2_ capture with the potential to enable negative emission technologies by maximizing mass transfer and CO_2_ affinity toward sorbent materials. Among diverse materials available, amine oligomers physically loaded in mesoporous supports are scalable and robust systems. Researchers have explored the role of support and amine composition and structure on the CO_2_ uptake performance, introduced additives, humidity, and altered operating conditions to gather new knowledge and promote understanding of these materials. However, less is known about the physical structure and mobility of the soft supported amine phase.

We chose PEI/SBA-15 systems as model sorbents due to their regular structure and the extensive literature data on these materials. Through a series of NS, ssNMR, and MD simulation studies, we learned that PEI/SBA-15 and similar amine/SBA-15 composites yield different amine structures and domains depending on the loading with some compositions dominated by an unfavorable distribution of amines. Amines impregnated into the solid supports form pore-coating domains driven by (typically) attractive wall-amine interactions. Consequently, a portion of the amines becomes nearly inactive toward CO_2_ capture, as they are tied around the pore walls. We further learned that attractive interactions between the wall and amines hamper amine mobility, thereby affecting CO_2_ diffusion through amine-loaded phases. Third, the presence of grafted chemical moieties on the pore walls brought about complex interplay between the native silica walls, chemically tethered groups, and PEI chains, which affected the amine structure and mobility and hence the CO_2_ sorption. Fourth, we observed that amines with different chain topologies exhibit unique distributions and mobility patterns that influence the diffusive properties of the amine molecules as well as CO_2_. Here are some key design principles based on the findings mentioned in this paper. First, retaining enough pore volume is crucial for the diffusion of CO_2_. Second, lessening the extent of pore-coating domains or making them easily turn from the walls can result in better CO_2_ uptake. This can be done by tuning the supports’ structures (e.g., structured supports with less extent of surface area per pore volume),^47,48^ pore surface chemistries, and the structures of aminopolymers. However, a careful approach should be made—too loosely bound amines or fast-moving amines may lessen materials’ stability.

We anticipate that the systematic experimental and simulation approach presented in this account will contribute to the formulation of more nuanced structure–property relationships, enabling a deeper understanding of these materials. We further envision that additional studies aimed at understanding the structures and dynamics of these materials will eventually lead to the development of a theoretical model or predictive framework for sorption and diffusion that can accelerate materials development.
